# Wilson disease complicated by Crohn disease: A case report and literature review

**DOI:** 10.1097/MD.0000000000033839

**Published:** 2023-06-16

**Authors:** Minmin Chen, Chenyan Li, Shiqiao Peng, Mengyuan Liu, Yiling Li, Mingjun Sun, Xuren Sun

**Affiliations:** a Department of Gastroenterology, First Affiliated Hospital of China Medical University, Shenyang, China; b Department of Endocrinology and Metabolism, First Affiliated Hospital of China Medical University, Shenyang, China; c Department of Gastrointestinal Endoscopy, First Affiliated Hospital of China Medical University, Shenyang, China.

**Keywords:** Crohn disease, hepatolenticular degeneration, inflammatory bowel disease, ustekinumab, Wilson disease

## Abstract

Wilson disease (WD), also known as hepatolenticular degeneration, is an autosomal-recessive hereditary disease with abnormal copper metabolism. Crohn disease (CD) is a chronic inflammatory gastrointestinal disease, which belongs to inflammatory bowel disease, all segments of the gastrointestinal tract can be affected, especially the terminal ileum and colon, accompanied by extraintestinal manifestations and related immune disorders. WD complicated by ulcerative colitis has been reported before, but WD complicated by CD has not been reported so far.

**Patient concerns and diagnosis:**

We presented the first report of a young patient with WD complicated by CD, who was admitted to the hospital because of repeated low fever, elevated C-reactive protein for 3 years, and anal fistula for 6 months.

**Interventions and outcomes:**

In this complicated disease, Ustekinumab is safe and effective.

**Lessons:**

We conclude that copper metabolism and oxidative stress play important roles in WD and CD.

## 1. Introduction

Wilson disease (WD) is a rare autosomal-recessive disease. Its pathogenesis is the failure of bile copper excretion caused by the mutation of the ATP7B gene and the inactivation of ATP7B transporter protein in hepatocytes, which leads to copper dynamic balance disorder. The most common symptoms are liver disease and neuropsychiatric disorders.^[1]^ Crohn disease (CD) is a chronic gastrointestinal inflammatory disease, which is believed to result from the interaction of genetic susceptibility, environmental factors, and intestinal microbes, leading to abnormal mucosal immune responses and impaired epithelial cell barrier function. Typical clinical presentations are abdominal pain, chronic diarrhea, weight loss, and fatigue in young patients.^[2]^ WD complicated by ulcerative colitis has been reported before, but WD complicated by CD has not been reported so far.^[3–6]^ We report the first case of WD complicated by CD and explore the possible association between the 2 diseases. Ustekinumab, a monoclonal antibody targeting the shared p40 subunit of IL-12/23, is approved for treatment of moderate-to-severe.^[7]^ In this complicated disease, Ustekinumab is safe and effective. We hope that this report elicits further studies exploring the mechanisms associating inflammatory bowel disease (IBD) and WD.

## 2. Case presentation

**Figure 1.**
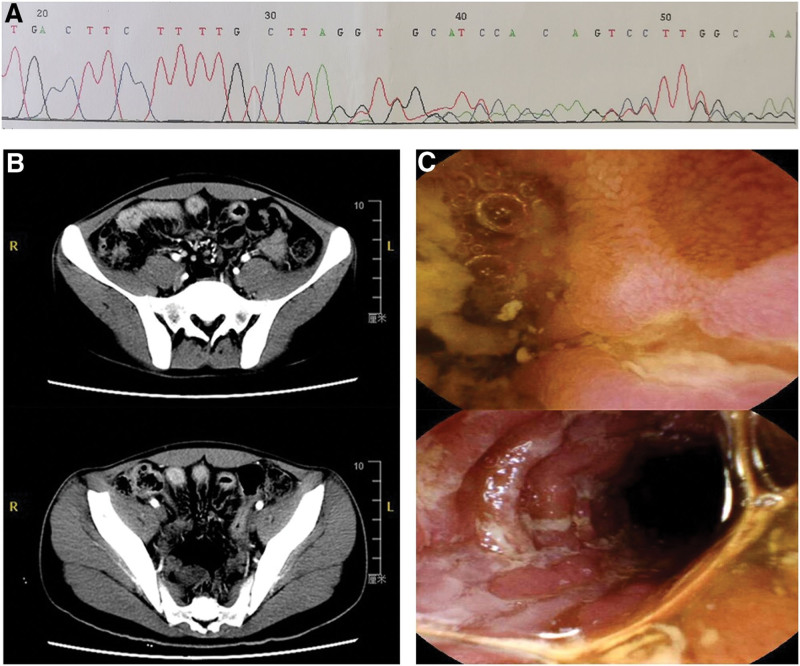
(A) ATP gene mutation screening in exon 1-21: a heterozygous mutation of 3700de1G was detected in exon 18. (B) Abdominal enhanced CT indicated that there was segmental thickening of the distal ileum wall, with the thickness approaching 1 cm, accompanied by significant enhancement, and “comb sign” of mesenteric vessels. (C) Capsule endoscopy revealed multiple, segmental, longitudinal fissure ulcers in the small intestine.

A 16-year-old boy was admitted to the first affiliated hospital of China Medical University because of repeated low fever, elevated C-reactive protein (CRP) for 3 years, and anal fistula for 6 months. At the age of 5, the patient was diagnosed with WD due to abnormal liver function. The diagnosis was based on detection of alanine transaminase 277 IU/L, aspartate transaminase 105 IU/L, urinary copper excretion 156 μg/d, ceruloplasmin 47.1 mg, and ATP gene mutation screening in exon 1-21: a heterozygous mutation of 3700de1G was detected in exon 18 (Fig. 1A). Since then, the patient has been treated with alternating oral administration of D-penicillamine and sodium dimercaptosulphonate (every 3 months), and the liver function returned to normal after 5 years. In the last 3 years, the patient has been complaining of persistent unknown causes of low fever, weight loss, and laboratory abnormalities, mainly characterized by increased CRP, increased erythrocyte sedimentation rate and elevated platelets, and the possibility of hematological diseases, tumors, and tuberculosis were ruled out. Six months ago, the patient developed an anal fistula. The fistula still didn’t heal after anorectal surgery. After admission, we started the examination of the entire digestive tract. The abdominal enhanced CT indicated that the multi-segmental wall of the small intestine was thickened with significant enhancement (Fig. 1B), and no colonic mucosal inflammation and destruction were found by colonoscopy. Capsule endoscopy revealed multiple, segmental, longitudinal fissure ulcers (Fig. 1C) in the small intestine. Laboratory examinations show ASCA59.8 Unit (+ reference value < 20.1), CRP57.6 mg/L, and calcitonin > 1800 μg/g, finally confirming the diagnosis CD.

**Figure 2.**
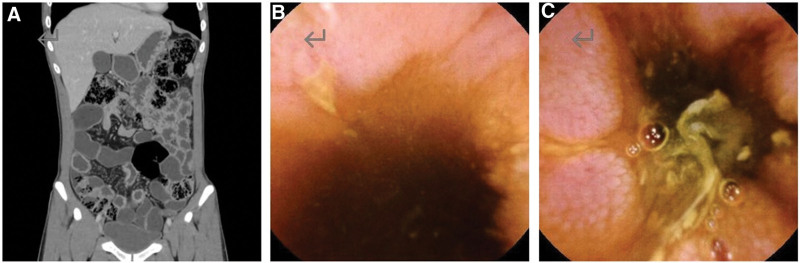
Reexamination of small intestine 3DCT and capsule endoscopy after 8 months of Ustekinumab therapy for Crohn disease, (A) small intestine 3D enhanced CT of the small intestine showed segmental thickening of the ileal wall, with uniform thickness and obvious enhancement. (B and C) Capsule endoscopy shows multiple, segmental, longitudinal fissure-like ulcers in the small intestine.

The patient refused to use infliximab for reasons of drug safety, medication mode, and privacy protection and actively chose ustekinumab. Before medication, the patient underwent relevant laboratory tests to exclude severe liver and kidney function damage, tumor, active infection, tuberculosis, hepatitis, and other diseases. The patient’s anal fistula gradually healed after medication, there was no fever, the patient’s CRP and erythrocyte sedimentation rate returned to normal, and his weight recovered. The patient continued Ustekinumab maintenance therapy with partial enteral nutrition, and no abnormal liver function was observed during treatment. CD and WD were evaluated 8 months after treatment: the patient gained 5 kg, was in better mental health, did not experience abdominal pain or diarrhea, pelvic MRI suggested healing of perianal lesions, and no abnormal inflammatory markers were detected. However, the reexamination of small intestinal 3DCT and capsule endoscopy still suggested the presence of multiple, segmental wall thickening and longitudinal fissure-like ulcers in the small intestine (Fig. 2). Compared with the time of diagnosis, after 8 months of treatment, the inflammatory activity of the patient relieved but did not achieve mucosal healing (Table 1). In the case of WD, there was no abnormality in liver function, nervous system, kidney, or eye examination. We finally maintained the Ustekinumab treatment and the original treatment for WD.

**Table 1:** Comparison of the effectiveness of Crohn disease before and after treatment.

Symptoms and signs laboratory examination	At diagnosis of Crohn disease	After 8 months of treatment
Diarrhea	Absent	Absent
Abdominal pain	Absent	Absent
Anal fistula	Present	Recovery
Nutritional status	Emaciation, fatigue, hypoproteinemia	Weight gain and normal nutritional status
Fever	Low fever	Normal
ALT and AST	Normal	Normal
CT manifestation	The multi-segmental wall of the small intestine was thickened with significant enhancement	3DCT: the multi-segmental wall of the small intestine was thickened with significant enhancement
CRP	225.2 mg/L	Normal
Calprotectin (<200)	>1800 μg/g	647 μg/g
Capsule endoscope	Multiple, segmental, longitudinal fissure-like ulcers in the small intestine.	Multiple, segmental, longitudinal fissure-like ulcers in the small intestine.
Kayser-Fleischer ring	Negative	Negative
Nervous system and renal function	Normal	Normal

ALT = alanine transaminase, AST = aspartate transaminase, CRP = C-reactive protein.

## 3. Discussion

WD is an autosomal-recessive hereditary disease, which is caused by a genetic defect in copper transporter ATP7B, thus resulting in pathological deposit of copper in the liver, brain, and other tissues.^[1,8]^ CD is one of the forms of IBD, the inflammation shows typically segmental, asymmetrical, transmural, or granulomatous features, which can affect any part of the gastrointestinal tract from mouth to perianal area.^[2,9]^ WD complicated by CD has not been reported so far. However, ulcerative colitis, another form of IBD complicating WD patients has been reported in several cases.^[3–6]^ Interestingly, our report, like most other cases, WD occurs prior to IBD, and our report undoubtedly increases the possibility of the correlation between WD and IBD.

As for the possible association between the 2 diseases, we continue the previous view in the case report of WD complicated by UC, namely intestinal inflammation and immune activation caused by abnormal copper metabolism. Oxidative stress has been demonstrated to be one of the pathophysiological bases of IBD.^[10,11]^ The imbalance between antioxidant defense and reactive oxygen species leads to oxidative stress, and reactive oxygen species promote chronic intestinal inflammation and immune activation by upregulating the production of pro-inflammatory cytokines, while copper is considered to act as a scavenger of oxygen free radicals in the inflammatory process.^[8,10–13]^ Takahiro Torisu concluded in his case report that the overloading of copper in tissues of patients with WD leads to excessive oxidative stress, which affects the liver and intestine and promotes inflammation.^[3]^ It is a known fact that there is evidence of high serum copper levels in patients with IBD.^[14,15]^ Therefore, there may be a correlation between high copper levels of WD and IBD. This was also mentioned in another case report, where the authors suggested that the onset of ulcerative colitis may also be the result of high levels of copper induced by WD.^[4]^ In addition, copper absorption in enterocytes is thought to be controlled by a plasma membrane copper transporter protein – Copper Transporter 1 (Ctr1).^[16]^ In eukaryotes (from yeast to humans), the Ctr1 family plays a key role in copper absorption across the plasma membrane.^[17]^ When Cu is overloaded in WD hepatocytes, the expression of Ctr1 appears to be down-regulated, and the down-regulation of Ctr1 expression may lead to increased Copper content in the intestine, further contributing to the deterioration of inflammation.^[17,18]^

Serum ceruloplasmin is a serum ferroxidase, which can limit myeloperoxidase activity in inflammatory sites, and be described as a protective shield against inflammation.^[19,20]^ As we all know, reduced serum ceruloplasmin is a characteristic of WD. There have been experiments showed that ceruloplasmin, as an acute phase plasma protein produced by hepatocytes and activated macrophages, was elevated in patients with IBD and was found in inflammatory infiltration and blood vessels, suggesting that it may have a protective effect on colitis due to its antioxidant activity.^[14,21]^ Clinical studies agree that lack of ceruloplasmin on its own is not sufficient to trigger colitis, but deficiency of ceruloplasmin, complete or nearly complete, will exacerbate the inflammation and accelerate the progression of colitis after other factors (such as toxicity, stress, or infection) trigger it.^[14,21,22]^ Combined with our case report, we make a conjecture that low serum ceruloplasmin caused by WD may be an aggravating factor in CD.

To sum up, we suggest that a correlation can be established between high Copper levels in CD and WD and that Ctr1 down-regulation and low serum ceruloplasmin may further aggravate CD. The symptom of WD in our patient was relatively mild, involving only the liver, and was well controlled by medication. Meanwhile, the onset of CD is insidious with slow progress, and it is difficult to diagnose which may be related to the control of WD. The correlation needs to be verified by more cases and experiments.

In terms of treatment, the current treatment of WD mainly includes drug therapy and liver transplantation. Drugs include chelating agents that increase urinary copper excretion (d-penicillamine and trientine), zinc salts that reduce copper absorption in the digestive tract and sodium dimercaptosulphonate. Liver transplantation is used to save patients with acute liver failure or who do not respond to drug treatment.^[23]^ Treatment for CD mainly relies on medication and surgery. However, surgery rarely cures CD patients, and drug therapy needs to be carried out simultaneously.^[9]^ Corticosteroids and immunosuppressants are traditionally used. But they are not suitable for long-term treatment due to their numerous adverse reactions. Antitumor necrosis factor is the earliest used biologic agent, among which infliximab was the first to be used for IBD and was widely used in patients with severe IBD.^[24]^ In this case, because the patient refused to use infliximab for reasons of drug safety, medication mode, and privacy protection, we finally chose ustekinumab for therapy, which has been shown to be both efficacious and safe in adult patients with CD. Ustekinumab is a monoclonal antibody IgG1 that blocks the P40 subunit of interleukin-12 (IL-12) and interleukin-23 (IL-23). It inhibits the inflammatory cascade through multiple pathways, thereby reducing systemic inflammation.^[25–27]^ Although its effectiveness in the pediatric population has not been reported, its off-label use in this age group is increasing, and more and more studies have shown that ustekinumab is effective and safe in pediatric patients with IBD.^[28,29]^ Additionally, it is worth mentioning that a clinical study showed that after applying Ustekinumab, 67% of antitumor necrosis factor refractory patients with perianal diseases showed improvement.^[30]^ However, to date, there is little data available on the effectiveness of Ustekinumab in perianal CD.^[25]^ For our patient, Ustekinumab showed a good therapeutic effect, not only improving the clinical symptoms but also curing the anal fistula. Although it accompanied WD, it did not cause the onset of WD, and there was no abnormality in liver function and various systems.

Overall, this is the first reported case of WD complicated by CD. Simultaneous occurrence of 2 rare diseases is very uncommon. This case increases the evidence of the association between WD and IBD, but more cases and basic research are needed in the future.

## 4. Conclusion

We reported the first case of WD complicated by CD. The attack of CD is considered to be the result of high levels of copper induced by WD, and Ctr1 down-regulation and low levels of serum ceruloplasmin may be an aggravating factor in CD. Ustekinumab can be a treatment option when WD is complicated by CD, especially in patients with anal fistula. And it is safe and effective even in children. However, more controlled clinical trial data are needed to confirm the clinical effectiveness and safety of ustekinumab in children and young adults. We hope that this case can provide new ideas for therapy of this combined disease and arouse people’s thinking and attention to the correlation between WD and CD.

## Author contributions

**Conceptualization:** Xuren Sun.

**Data curation:** Minmin Chen.

**Formal analysis:** Chenyan Li, Shiqiao Peng, Mengyuan Liu.

**Project administration:** Yiling Li, Mingjun Sun.

**Supervision:** Yiling Li, Mingjun Sun, Xuren Sun.

**Visualization:** Chenyan Li, Shiqiao Peng, Mengyuan Liu.

**Writing – original draft:** Minmin Chen.

**Writing – review & editing:** Minmin Chen, Xuren Sun.
